# Socioeconomic Position Disparities in Cardiovascular Health Before and After the Examination of Mechanisms of Exercise-Induced Weight Compensation Randomized Controlled Trial

**DOI:** 10.1089/heq.2019.0019

**Published:** 2019-08-07

**Authors:** Candice A. Myers, Stephanie T. Broyles, Corby K. Martin

**Affiliations:** Department of Population & Public Health Sciences, Pennington Biomedical Research Center, Baton Rouge, Louisiana.

**Keywords:** socioeconomic position, health disparity, cardiovascular health, exercise intervention

## Abstract

**Purpose:** We examined socioeconomic position (SEP) disparities in cardiovascular health before and after an exercise intervention.

**Methods:** Data were from the Examination of Mechanisms of Exercise-Induced Weight Compensation (E-MECHANIC) study. Cardiovascular health was measured through a composite score combining body mass index, systolic blood pressure, cholesterol, and glucose. SEP was assessed using a single measure that combined income and education.

**Results:** At baseline, there was no significant difference in cardiovascular health between high and low SEP participants. Post-intervention, this difference reached significance.

**Conclusion:** Although cardiovascular health improved for exercise intervention participants, SEP disparities in cardiovascular health persisted during the trial.

## Introduction

Cardiovascular disease is the leading cause of death in the United States.^1^ Research into the factors that influence cardiovascular health has shown that social determinants are relevant for understanding cardiovascular disease risks and outcomes.^2^ Social determinants are the conditions in which people are born, grow, live, work, and age.^3^ Havranek et al. identified six social determinants of cardiovascular health, including socioeconomic position (SEP), which is often measured through income, education, and employment/occupation to capture the economic resources available to an individual.^2^ Importantly, a large body of research has shown SEP disparities in cardiovascular disease and mortality, with higher and lower SEP being associated with better and worse cardiovascular health, respectively.^2,4–8^

Health disparities associated with SEP hold implications for interventions by potentially influencing heterogeneous response among participants. A number of intervention-based studies targeting health improvement have assessed intervention effectiveness according to SEP. Focusing on cardiovascular health, Govil et al.^9^ examined the effectiveness of the Multisite Cardiac Lifestyle Intervention Program between low and high socioeconomic status (SES) participants. Although they saw differences in coronary risk factors at the beginning of the study, after the intervention the researchers did not witness differences in response to the intervention between low and high SES participants with both groups improving in coronary risk factors. Similar questions have been investigated in interventions targeting diabetes and obesity prevention. In the Diabetes Prevention Program (DPP), Wing et al.^10^ examined the effect of socioeconomic measures, including income, on weight loss response to the DPP lifestyle intervention and found no significant influence of socioeconomic variables on a participant's ability to achieve weight loss. The Programme for the Prevention of Type 2 Diabetes in Finland (FIN_D2D) was a lifestyle intervention targeting diabetes prevention.^11^ Rautio et al.^11^ found that SEP did not influence the effect of the FIN_D2D intervention with equivalent response in all SEP groups. However, after the Special Diabetes Program for Indians Diabetes Prevention (SDPI_DP), Jiang et al.^12^ found SES disparities in weight outcomes after the intervention. In their review, Beauchamp et al.^13^ reported mixed findings regarding the effectiveness of obesity prevention interventions between different SEP groups, with studies being both effective and ineffective in lower SEP participants.

Above and beyond intervention effectiveness, it is also important to understand how interventions impact SEP health disparities. Although interventions may achieve health improvements in participants of varying SES, SEP health disparities may persist in the context of an intervention and, in turn, an effective intervention can maintain or increase SEP inequities in the health outcome of interest.^14,15^ Given this, the objective of this study was to investigate how an intervention impacted SEP disparities in cardiovascular health. This objective was achieved by examining SEP-based differences in cardiovascular health before and after the Examination of Mechanisms of Exercise-Induced Weight Compensation (E-MECHANIC) exercise trial.

## Methods

### Participants

Data were drawn from the E-MECHANIC randomized controlled exercise trial (ClinicalTrials.gov ID: NCT01264406). The full details of the trial design and primary results are published elsewhere.^16,17^ In brief, E-MECHANIC tested the effect of exercise on energy intake and body weight by randomizing 198 sedentary participants with overweight or obesity in a 1:1:1 ratio with (1) a nonexercise control group or one of two exercise conditions that reflected exercise guidelines for (2) general health (8 kcal/kg body weight per week [8 KKW]) or (3) weight loss (20 KKW) for 6 months. The nonexercise control group received health information only. The 8 KKW exercise group completed their full exercise dose from the beginning of the 6 months. The 20 KKW exercise group ramped up their exercise dose from 8 KKW during week 1 to 14 KKW during week 2 and 20 KKW during week 3. By the end of the third week, participants performed 100% of their weekly exercise dose for 3–4 days in the 8 KKW group and for 3–5 days in the 20 KKW group. All exercise training occurred on a treadmill and all exercise sessions were monitored/supervised on-site at Pennington Biomedical Research Center (PBRC) by study staff.

For this study, we analyzed data for those participants (*n*=133) who received either of the two exercise doses to focus on response in cardiovascular health to a prescribed, structured, and tightly controlled exercise intervention. All study procedures and secondary data analyses were approved by the PBRC Institutional Review Board and all participants provided written informed consent.

### Measures

#### Dependent variable: cardiovascular health

The American Heart Association (AHA) set national goals for cardiovascular health, including seven metrics for ideal cardiovascular health that encompass both health behaviors and health factors.^18^ For this study, we measured four AHA cardiovascular health metrics: body mass index, systolic blood pressure, cholesterol, and glucose. We created a composite cardiovascular health score based upon ideal (2 points), intermediate (1 point), and poor (0 points) health for each metric for a possible range of 0–8 points (worst to best cardiovascular health).^19^ These scores were calculated at baseline and postintervention.

#### Primary exposure: SEP

We measured SEP using self-reported income and level of education. Income was reported in increments of $20,000 or $30,000 from <$10,000 to >$130,000. Educational attainment was reported as some high school, high school diploma/General Educational Development (GED), 1–3 years of college, college degree, or postgraduate degree. To assess the simultaneous influence of multiple indicators of SEP,^2^ we created a single factor score using principal components analysis with income and education, controlling for race. We next categorized participations as high SEP (=1) or low SEP (=0) according to whether their SEP factor score fell above (high SEP) or below (low SEP) 0.

### Statistical analysis

We regressed cardiovascular health scores against SEP in a linear repeated-measures mixed model, controlling for gender, age, marital status, and race, using Kenward–Roger approximation to calculate denominator degrees of freedom for fixed effect tests (SAS version 9.4, PROC MIXED).^20^ We included an interaction term between time (baseline vs. postintervention) and SEP to examine differences of least squares means (LS-means), which were adjusted for multiple comparisons (Tukey–Kramer). *p*-Value <0.05 was used to determine significance. The final analytic sample consisted of 115 participants who received the E-MECHANIC exercise intervention and had no missing data for all variables.

## Results

**FIG. 1.**
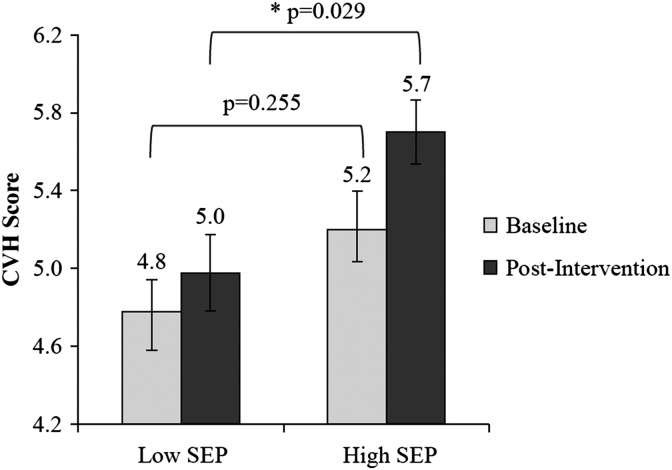
Cardiovascular health between low SEP and high SEP participants before and after the E-MECHANIC exercise intervention. E-MECHANIC, Examination of Mechanisms of Exercise-Induced Weight Compensation; SEP, socioeconomic position.

Participants were largely female and married with an average age of 48 years and 30% of the sample was African American (Table 1). The mean cardiovascular health factor score at baseline was 5.1 points (standard deviation [SD]=1.2 points) and 5.4 points (SD=1.2 points) post-intervention. Regression analysis revealed that the exercise intervention increased cardiovascular health overall during the 6-month trial (*p*<0.001). The time-by-SEP interaction term was not significant (*p*=0.160), indicating that the E-MECHANIC exercise intervention effectively increased cardiovascular health for both high and low SEP participants. At baseline, participants in the high SEP group had higher cardiovascular health scores, although the difference was not significant (LS-means difference=0.44; *p*=0.255). However, after the exercise intervention, the difference in cardiovascular health scores between high and low SEP participants not only increased but also reached significance (LS-means difference=0.67; *p*=0.029) (Fig. 1).

**Table 1.** Participant Characteristics and Cardiovascular Health from the Examination of Mechanisms of Exercise-Induced Weight Compensation Exercise Intervention

Variables	Total	High SEP	Low SEP
*n*	115	65	50
Female, *n* (%)	83 (72)	42 (65)	41 (82)^*^
Age, mean (SD) [range]	48 (11.7) [21–65]	49 (10.9) [21–65]	48 (12.7) [25–65]
Married, *n* (%)	69 (60)	49 (75)	20 (40)^*^
African American, *n* (%)	34 (30)	20 (31)	14 (28)
SEP			
Income, *n* (%)			
<$10,000	2 (2)		2 (4)
$10,000–$29,999	12 (10)		12 (24)
$30,000–$49,999	15 (13)	2 (3)	13 (26)
$50,000–$79,999	30 (26)	13 (20)	17 (34)
$80,000–$99,999	20 (17)	14 (22)	6 (12)
$100,000–$129,999	18 (16)	18 (28)	
>$130,000	18 (16)	18 (28)	
Education, *n* (%)			
Some high school	1 (1)		1 (2)
High school diploma/GED	9 (8)		9 (18)
1–3 Years college	29 (25)	10 (15)	19 (38)
College degree	46 (40)	26 (40)	20 (40)
Postgraduate degree	30 (26)	29 (45)	1 (2)
CVH score, mean (SD) [range]			
Baseline	5.1 (1.2) [2–7]	5.2 (1.3) [2–7]	5.0 (1.2) [2–7]
Post-intervention	5.4 (1.2) [2–8]	5.6 (1.1) [3–8]	5.2 (1.2) [2–7]^*^

^*^ *p*<0.05, significant difference between high SEP and low SEP.

SD, standard deviation; SEP, socioeconomic position.

## Conclusion

This study found that the E-MECHANIC exercise intervention effectively improved cardiovascular health for participants regardless of SEP. However, our analysis did reveal that differences in cardiovascular health between high and low SEP participants maintained over the course of the intervention with high SEP participants having significantly better cardiovascular health than low SEP participants after the trial. Although other studies and reviews have found mixed evidence regarding the impact of SEP on intervention effectiveness,^9–13^ our results highlight the role of SEP and its impact on cardiovascular health disparities in the context of an intervention. That is, interventions aimed at improving cardiovascular health can successfully achieve this goal, but can also maintain and even widen socioeconomic health disparities.

We point out that the E-MECHANIC trial was not a lifestyle intervention designed to improve cardiovascular health through physical activity (i.e., exercise), but instead examined the mechanisms of weight compensation during an exercise trial, which is both a strength and weakness of this study. The E-MECHANIC trial provided a unique opportunity to examine whether SEP was associated with differential intervention response in cardiovascular health, as well as how SEP-based health disparities operated during a structured and tightly controlled intervention with a prescribed exercise dose that was supervised. Our study identified the influence of SEP as a social determinant of health and source of health disparities even within the context of a randomized controlled exercise trial with excellent adherence.^17^

This study holds important implications for intervention-based research targeting cardiovascular health, among other relevant health outcomes. Although interventions can effectively induce improvements in cardiovascular health to varying degrees across socioeconomic strata, there exists the potential for such interventions to maintain and increase cardiovascular health disparities. This point has been documented by other health researchers who utilize the terminology “intervention-generated inequalities” to describe health inequalities driven by interventions, particularly health inequities resulting from SEP.^14,21^ Our research further fits within this body of literature and its call to examine the effectiveness and equity of interventions through critical comparative analysis to better identify and prioritize those strategies that not only induce health improvement but also have equitable impact.^13,14^ In order for interventions to not only be effective but also avoid creating and perpetuating health inequalities, consideration must be given to the role of socioeconomic health disparities in intervention development to close the gap between more and less advantaged groups when it comes to health improvement. As demonstrated here, factors that are external or not directly impacted by an intervention, such as SES and related factors, can still assert influence and shape disparate intervention response.

## Abbreviations Used

AHA: American Heart Association
DPP: Diabetes Prevention Program
E-MECHANIC: Examination of Mechanisms of Exercise-Induced Weight Compensation
PBRC: Pennington Biomedical Research Center
SD: standard deviation
SEP: socioeconomic position
SES: socioeconomic status

## References

[1] Murphy SL, Xu J, Kochanek KD, et al. Mortality in the United States, 2017 Hyattsville, MD: National Center for Health Statistics, 2018

[2] Havranek EP, Mujahid MS, Barr DA, et al. Social determinants of risk and outcomes for cardiovascular disease: a scientific statement from the American Heart Association. Circulation. 2015;132:873–898 26240271 10.1161/CIR.0000000000000228

[3] World Health Organization. About Social Determinants of Health. Available at www.who.int/social_determinants/sdh_definition/en Accessed 65, 2018

[4] Wolfe B, Evans W, Seeman TE The Biological Consequences of Socioeconomic Inequalities. New York: Russell Sage Foundation, 2012

[5] Kaplan GA, Keil JE Socioeconomic factors and cardiovascular disease: a review of the literature. Circulation. 1993;88(4 Pt 1):1973–1998 8403348 10.1161/01.cir.88.4.1973

[6] Mensah GA. Eliminating disparities in cardiovascular health: six strategic imperatives and a framework for action. Circulation. 2005;111:1332–1336 15769777 10.1161/01.CIR.0000158134.24860.91

[7] Mensah GA, Mokdad AH, Ford ES, et al. State of disparities in cardiovascular health in the United States. Circulation. 2005;111:1233–1241 15769763 10.1161/01.CIR.0000158136.76824.04

[8] Martinson ML, Teitler JO, Plaza R, et al. Income disparities in cardiovascular health across the lifespan. SSM Popul Health. 2016;2:904–913 29349197 10.1016/j.ssmph.2016.10.009 PMC5757909

[9] Govil SR, Weidner G, Merritt-Worden T, et al. Socioeconomic status and improvements in lifestyle, coronary risk factors, and quality of life: the Multisite Cardiac Lifestyle Intervention Program. Am J Public Health. 2009;99:1263–1270 18923113 10.2105/AJPH.2007.132852 PMC2696652

[10] Wing RR, Hamman RF, Bray GA, et al. Achieving weight and activity goals among diabetes prevention program lifestyle participants. Obes Res. 2004;12:1426–1434 15483207 10.1038/oby.2004.179 PMC2505058

[11] Rautio N, Jokelainen J, Oksa H, et al. Socioeconomic position and effectiveness of lifestyle intervention in prevention of type 2 diabetes: one-year follow-up of the FIN-D2D project. Scand J Public Health. 2011;39:561–570 21622677 10.1177/1403494811408482

[12] Jiang L, Huang H, Johnson A, et al. Socioeconomic disparities in weight and behavioral outcomes among American Indian and Alaska Native Participants of a Translational Lifestyle Intervention Project. Diabetes Care. 2015;38:2090–2099 26494807 10.2337/dc15-0394 PMC4613924

[13] Beauchamp A, Backholer K, Magliano D, et al. The effect of obesity prevention interventions according to socioeconomic position: a systematic review. Obes Rev. 2014;15:541–554 24629126 10.1111/obr.12161

[14] White M, Adams J, Heywood P. How and why do interventions that increase health overall widen inequalities within populations? In: Social Inequality and Public Health. Babones S (Ed). Bristol, United Kingdom: Policy Press, 2009, pp. 64–81

[15] McGill R, Anwar E, Orton L, et al. Are interventions to promote healthy eating equally effective for all? Systematic review of socioeconomic inequalities in impact. BMC Public Health. 2015;15:457 25934496 10.1186/s12889-015-1781-7 PMC4423493

[16] Myers CA, Johnson WD, Earnest CP, et al. Examination of mechanisms (E-MECHANIC) of exercise-induced weight compensation: study protocol for a randomized controlled trial. Trials. 2014;15:212 24906459 10.1186/1745-6215-15-212 PMC4057557

[17] Martin CK, Johnson WD, Myers CA, et al. Effect of different doses of supervised exercise on food intake, metabolism, and non-exercise physical activity: the E-MECHANIC randomized controlled trial. Am J Clin Nutr. 2019;108:1–10 10.1093/ajcn/nqz054 PMC6735935 31172175

[18] Lloyd-Jones DM, Hong Y, Labarthe D, et al. Defining and setting national goals for cardiovascular health promotion and disease reduction: the American Heart Association's strategic Impact Goal through 2020 and beyond. Circulation. 2010;121:586–613 20089546 10.1161/CIRCULATIONAHA.109.192703

[19] Thacker EL, Gillett SR, Wadley VG, et al. The American Heart Association Life's Simple 7 and incident cognitive impairment: the REasons for Geographic And Racial Differences in Stroke (REGARDS) study. J Am Heart Assoc. 2014;3:e000635 24919926 10.1161/JAHA.113.000635 PMC4309046

[20] Kenward MG, Roger JH Small sample inference for fixed effects from restricted maximum likelihood. Biometrics. 1997;53:983–997 9333350

[21] Lorenc T, Petticrew M, Welch V, et al. What types of interventions generate inequalities? Evidence from systematic reviews. J Epidemiol Commun Health. 2013;67:190–193 10.1136/jech-2012-201257 22875078

